# From person-specific networks to personalized psychiatry: What evidence is still needed?

**DOI:** 10.3389/fpsyt.2026.1967025

**Published:** 2026-09-17

**Authors:** Zheng Zhang

**Affiliations:** Department of Psychology, South China Normal University, Guangzhou, China

**Keywords:** clinical utility, ecological momentary assessment, idiographic analysis, network-informed intervention, personalized psychiatry, person-specific networks, prediction

## Introduction

Individual differences remain a central challenge in psychiatry: patients with the same diagnosis can differ in symptoms, trajectories, and treatment response, so group averages need not characterize an individual. Intensive longitudinal assessment and person-specific network analysis use ecological momentary assessment (EMA) to estimate contemporaneous and temporal relations within individuals (1, 2).

Person-specific network studies span mental health problems but vary in sampling density, duration, node selection, model choice, and assumption checking (3). These networks are statistical models, not clinical tools. Personalized psychiatry requires evidence that they improve prediction, decisions, and patient outcomes, consistent with contextualized, dynamic accounts of mental disorder (4, 5).

Estimating a network from one person’s data establishes individualized estimation, not necessarily personalization. Accordingly, we use “reliable” for an estimable model with reproducible dynamics (Levels 1-3), “informative” for incremental out-of-sample prediction (Level 4), and “useful” for effects on decisions or outcomes (Levels 5-6); the Personalization Ladder formalizes these distinctions below.

The Ladder therefore separates estimation, validation, prediction, decision support, and outcome improvement. The Personalization Ladder is illustrated in **Figure 1**.

**Figure 1:**
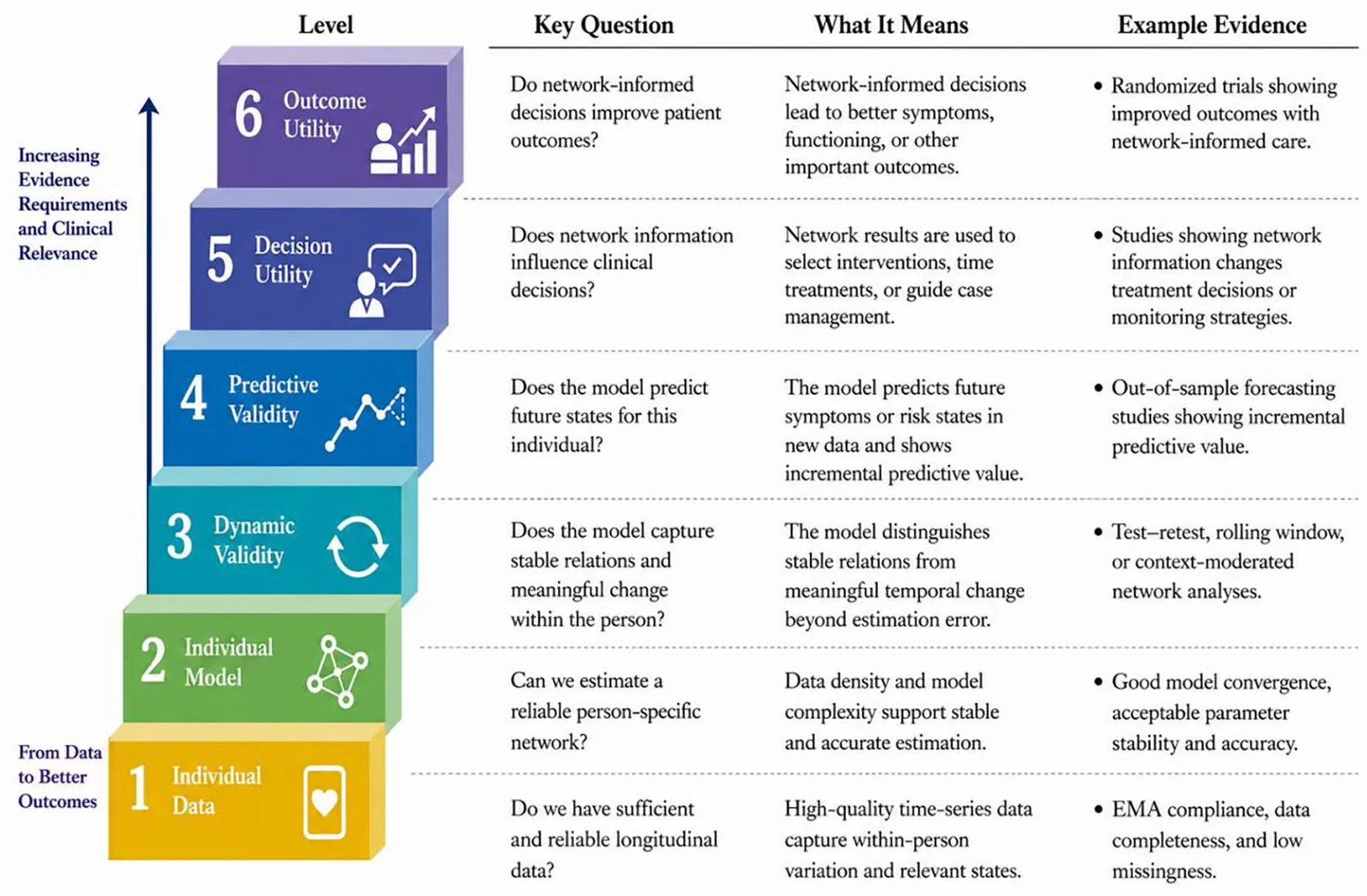
The personalization ladder. The six levels distinguish individual data and modeling from dynamic, predictive, decision, and outcome utility. Evidentiary requirements and clinical relevance increase from Levels 1 to 6; the Ladder is an evaluative framework rather than a mandatory linear development sequence.

## What makes a network genuinely person-specific?

Because a person-specific network is estimated within a finite observation period, repeated reports may reflect either enduring relations or processes specific to that period.

Relations may change during medication adjustment, psychotherapy, life events, or social-context shifts; moderated idiographic models can represent such context dependence (6). The key question is whether variation reflects genuine process change or estimation error.

Finite data also constrain temporal networks: autoregressive and cross-lagged parameters multiply as nodes are added, creating the ‘big network, short time series’ problem (7). Model complexity must match the number of valid observations.

Operationally, Level 1 should require a prespecified data-quality plan. An illustrative target is at least 80% completed prompts, with reporting of valid lagged observations, item/time-specific missingness, and sufficient within-person variation. Level 2 should require convergence and simulation-based recovery for the planned node-to-time-point ratio. VAR studies should justify precision using power or predictive-accuracy analyses and prespecify a clinically meaningful accuracy target; no universal R-squared cutoff is assumed (7).

At Level 3, comparable windows should show at least 80% sign agreement for prespecified key edges or an intraclass correlation of at least.60 for prespecified summary parameters, as appropriate. Claimed change should exceed bootstrap or joint-estimation uncertainty and persist across adjacent windows or a prespecified moderator. These are pragmatic floors, not universal constants.

## Does the individual model add information beyond group knowledge?

Individual heterogeneity does not imply that every patient needs a fully independent model. The clinically relevant question is whether person-specific estimation adds information beyond group models and routine measures.

Jover Martínez and colleagues found that group-level models described many participants reasonably well, while a subset was better represented by person-specific models; generalizability also depended on regularization (8). Model class is therefore part of the evidentiary claim. Separate VARs prioritize idiographic fit but may be unstable; GVAR/mlVAR borrow group information; regularized models reduce variance while altering edge selection; and moderated models allow context-dependent parameters (6, 8). Each rung should be evaluated for a model-target pair, with sensitivity analyses across plausible lag structures, regularization, and pooling assumptions. Hoekstra and colleagues likewise caution that a plotted network is only one representation of underlying dynamics (9).

Incremental value should be tested directly against group models, current symptom severity, clinical history, and simple autoregressive baselines. Individualization is compelling when it identifies reproducible information that these alternatives miss.

Network heterogeneity is relatively easy to demonstrate. The harder question is which differences are stable and practically useful.

## Prediction provides a stronger test of personalization

Estimated structure can describe recent dynamics, but personalized psychiatry is concerned with what happens next. Prediction therefore provides a stronger external test than identifying strong edges or apparently central symptoms.

At Level 4, evaluation should use temporally ordered holdout data or rolling-origin validation. As an illustrative floor, a study could preregister at least a 5% reduction in MAE or RMSE, or an equivalent proper-score gain, over persistence or autoregressive baselines, with an uncertainty interval excluding no improvement; calibration should also be reported. The threshold should be replaced when a condition-specific clinical or decision-analytic target is available.

Fechtelpeter and colleagues compared several model classes in micro-randomized trial data and found meaningful differences in forecasting spontaneous and intervention-related EMA changes (10). Similar distinctions apply to digital phenotyping: individualized prediction may be feasible, but it must be shown to improve a clinical decision rather than merely fit passively collected data. The broader lesson is that interpretability alone does not establish personalization; models must carry reproducible information about future states.

Patient-perceived accuracy may still support formulation and communication. Subjective evaluations differ across idiographic networks (9), but face validity and forecasting performance answer different questions and should be reported separately.

## Clinical personalization requires evidence of utility

Prediction alone does not establish clinical benefit. For psychotherapy, von Klipstein and colleagues describe person-specific networks as aids to collaborative case formulation, while emphasizing that formulation support is not evidence of causal mechanism or treatment efficacy (11). At Level 5, network information must enter a decision process and change treatment selection, timing, sequencing, monitoring, or case management in a prespecified, auditable way.

Qu and colleagues provide a second translational example: they used 10-day EMA networks to prioritize digital intervention modules (12). Symptoms improved in both network-informed and standardized-sequence groups, but the pilot found no clear additional benefit; the imprecise between-group estimate is inconclusive rather than evidence of equivalence. The study therefore illustrates Level 5 decision use without establishing Level 6 outcome utility.

Level 5 studies should report how often recommendations change care, clinician and patient uptake, decision time, and unintended consequences. Level 6 requires a randomized or credible pragmatic comparison showing improvement that meets a prespecified minimal clinically important difference, efficiency threshold, or net-benefit criterion without unacceptable harms.

## A Personalization Ladder

We propose the Personalization Ladder as a progressive evidential framework.

Level 1 is individual data of sufficient density and quality. Level 2 is an estimable and recoverable individual model. Level 3 is dynamic validity: stable relations are distinguished from meaningful change and estimation uncertainty. Level 4 is predictive validity in new observations, including incremental value over simple baselines. Level 5 is decision utility: network information changes management. Level 6 is outcome utility: those changes improve symptoms, functioning, efficiency, or other patient-important outcomes.

The provisional criteria above make the lower and middle rungs auditable. For Levels 5-6, universal effect-size cutoffs would be misleading because consequences vary by decision and disorder; studies should instead preregister the smallest change that would alter care or meet a patient-important outcome threshold.

Qu et al. provide a worked example (12). Their study collected individual EMA data (Level 1), estimated person-specific networks (Level 2), and used those networks to sequence modules (Level 5). Evidence for stability and out-of-sample forecasting was not reported as a prerequisite (Levels 3-4), and the pilot did not establish incremental outcome benefit (Level 6). The Ladder therefore identifies both the study’s genuine translational advance and the evidence still needed.

The ladder is evidential rather than a mandatory development sequence: a study may test an upper rung directly, but missing lower-rung evidence limits interpretation of why it succeeded or failed. Methodological studies can appropriately claim person-specific modeling at Levels 1-3; stronger claims about individualized prediction or personalized psychiatry require evidence at Levels 4-6.

The purpose is not to create a universal certification score, but to make claims testable. The numerical criteria above are transparent, provisional design targets and should be revised as empirical benchmarks mature.

## Discussion

Person-specific networks have made within-person temporal processes a direct object of psychopathology research. The next challenge is to establish when those models are reproducible, predictive, and clinically useful.

Burden and equity are part of that evidence, not implementation afterthoughts. Smartphone and data access, digital literacy, language, disability, symptom severity, caregiving or employment demands, and privacy concerns can affect EMA participation. Meta-analytic evidence places average EMA compliance near 80% and shows that incentives and design choices can influence adherence (13). Missingness may therefore be socially patterned, excluding people for whom personalized care is most needed. Studies should provide devices or data support where necessary, use accessible multilingual and low-bandwidth options, allow flexible schedules, compensate participation, offer non-smartphone alternatives, and report compliance, missingness, usability, and model performance across relevant demographic and clinical groups.

At Levels 5-6, governance responsibility should also be explicit. Developers are responsible for analytic validation and change control; health systems for local validation, workflow integration, and monitoring; and clinicians for contextual judgment, documented override, and communication with patients. Depending on intended use and jurisdiction, regulators and institutional oversight bodies may also be involved. Validation plans should specify data stewardship, consent, transparency, human oversight, audit trails, liability, adverse-event reporting, subgroup performance, and post-deployment drift. These requirements align with WHO principles on autonomy, accountability, inclusiveness, and equity, and with lifecycle guidance for machine-learning-enabled medical devices (14, 15).

Person-specific networks already have clear value for studying individual dynamics. Related precision-psychiatry work in individual-specific functional neuroanatomy illustrates that individualized representation can extend beyond symptom networks, while still requiring reliability and clinical validation (16). The role of person-specific networks in personalized psychiatry will depend on whether gains in prediction, decisions, and outcomes justify the measurement burden and are distributed equitably (17).

The Ladder also has limitations. The proposed thresholds are pragmatic rather than definitive, and different disorders, outcomes, and clinical settings may require different evidentiary standards. Future work should empirically calibrate these criteria through multi-site studies and prospective implementation research.
